# Multi-AOP: a lightweight multi-view deep learning framework for antioxidant peptide discovery

**DOI:** 10.1186/s40643-025-01004-1

**Published:** 2026-02-02

**Authors:** Jianxiu Cai, Xinpo Lou, Chak Fong Chong, Deepa Alex, Joel P. Arrais, Yapeng Wang, Shirley W. I. Siu

**Affiliations:** 1 Faculty of Applied Sciences, Macao Polytechnic University, Rua de Luís Gonzaga Gomes, Macau SAR, China; 2 Department of Informatics Engineering, University of Coimbra, Paço das Escolas, Coimbra, Portugal; 3 Centre for Artificial Intelligence Driven Drug Discovery, Macao Polytechnic University, Rua de Luís Gonzaga Gomes, Macau SAR, China; 4 BioMyne-Biotech Innovation and Engineering Ltd, Macau SAR, China

## Abstract

Antioxidant peptides (AOPs), with their strong free radical scavenging ability and health benefits, have emerged as promising candidates for disease prevention and food preservation. However, traditional experimental approaches to AOP discovery remain hindered by inefficiencies and substantial resource demands. Here, we present Multi-AOP, a parameter lightweight multi-view deep learning framework (0.75 million parameters) that enhances AOP discovery through integrated sequence and graph learning. We employ Extended Long Short-Term Memory (xLSTM) to generate sequence embeddings. Concurrently, we transform peptide sequences into SMILES representations and extract molecular graph features using a Message Passing Neural Network (MPNN), capturing intrinsic physicochemical properties. By leveraging both sequence patterns and structural information through hierarchical fusion, Multi-AOP achieves accuracies of 0.8043, 0.9684, and 0.9043 on the AnOxPePred, AnOxPP, and AOPP benchmark datasets, respectively, consistently outperforming conventional machine learning algorithms and state-of-the-art deep learning approaches. Furthermore, we constructed a unified AOP dataset by integrating these benchmark datasets, facilitating the future development of generalizable AOP models. All datasets and the optimized predictive model are publicly accessible at https://github.com/CaiJianxiu/Multi-AOP.

**Supplementary Information:**

The online version contains supplementary material available at 10.1186/s40643-025-01004-1.

## Introduction

Reactive oxygen species (ROS) are generated during cellular metabolism and are normally neutralized by the antioxidant defense system (Apel and Hirt 2004). However, excess ROS compromise redox homeostasis, cause tissue damage, and contribute to chronic diseases (Lian-Jiu et al. 2019; Jomova et al. 2023). Oxidative damage also affects food quality (Choe and Min 2005), pharmaceutical stability (Saravanakumar et al. 2017), and cosmetic shelf-life (Othman et al. 2020)

While synthetic antioxidants are effective, concerns over their potential toxicity have prompted a shift towards natural alternatives (Pop et al. 2013; Khezerlou et al. 2022). Antioxidant peptides (AOPs) have emerged as promising candidates due to their free radical-scavenging activity and additional bioactive properties (Elias et al. 2008). However, traditional experimental approaches–including chemical assays, cell-based methods, and *in vivo* models (Irina Georgiana Munteanu and Constantin Apetrei 2021)–remain time-intensive and resource-demanding. This limitation has driven the development of computational methods.

Recent advances in deep learning have driven significant progress in bioactive peptide prediction. For antimicrobial peptides, architectures such as recurrent neural networks (Velickovic et al. 2017) and attention-based models (Yan et al. 2023; Cai et al. 2025; Li et al. 2025) have achieved strong performance. Transfer learning using pre-trained protein language models (PLMs), including ProtBERT (Elnaggar et al. 2021) and ESM (Lin et al. 2022), has demonstrated effectiveness across multiple peptide classification tasks. Graph neural networks (GNNs) have also been applied to capture molecular structural information (Gilmer et al. 2020). More recently, hybrid frameworks combining sequence and graph representations, such as GraphPep (Tao et al. 2025) and Multi-peptide (Badrinarayanan et al. 2024), have shown promise for peptide property prediction.

For AOP prediction specifically, Olsen et al. developed AnOxPePred using convolutional neural networks (CNNs) with one-hot encoding (Olsen et al. 2020). Qin et al. implemented a bidirectional LSTM with amino acid descriptors (Qin et al. 2023), and Li et al. developed AOPP integrating multiple sequence features (Li et al. 2025). Despite this progress, existing AOP predictors predominantly rely on sequence composition and handcrafted features, neglecting structural information that influences peptide bioactivity. This limitation motivates multi-view learning approaches that integrate complementary feature representations to improve predictive performance and generalizability.

In this work, we introduce Multi-AOP, a parameter lightweight multi-view framework that integrates sequence embeddings from an xLSTM with molecular-graph features from a Message Passing Neural Network (MPNN). Unlike existing hybrid approaches that typically employ large PLMs, our framework uses a parameter-efficient xLSTM and MPNN backbone ( 0.75M parameters) suitable for limited AOP training data, combined with a hierarchical fusion strategy that effectively integrates sequence and structural features. We also provide a unified AOP dataset combining three public benchmarks to facilitate future model development.

## Materials and methods

### Dataset

We collected three distinct existing datasets as the benchmark datasets: the AnOxPePred dataset from Olson et al., the AnOxPP dataset from Qin et al., and the AOPP dataset from Li et al. The AnOxPePred dataset, sourced from the BIOPEP-UWM database in 2020, comprises 676 free radical scavenger (FRS) AOP as positive samples and 728 non-AOPs as negative samples. The negative set includes 218 experimentally validated non-AOP supplemented with 500 randomly generated sequences. The AnOxPP dataset, compiled in 2023 from the DFBP and BIOPEP-UWM databases, contains 1060 peptides with documented radical scavenging activities as positive samples, balanced with 1060 randomly generated sequences serving as negative samples. The most recent AOPP dataset collected in 2025 represents the most comprehensive collection. AOPP integrated data from multiple repositories including DFBP, BIOPEP-UWM, Antimicrobial Peptide Database, PlantPepDB, and FermFooDb. This dataset encompasses 1511 validated AOPs and an equivalent number of randomly generated sequences as negative controls.BIOPEP-UWM contains information on peptide sequences, source proteins, and biological activities. DFBP provides functional annotations including activity types and source organisms. The Antimicrobial Peptide Database includes peptide properties such as length, net charge, and hydrophobicity. PlantPepDB focuses on plant-derived peptides with source organism information. FermFooDB contains peptides derived from fermented foods with associated functional properties. A summary of the three benchmark datasets is provided in Table 1.

**Fig. 1:**
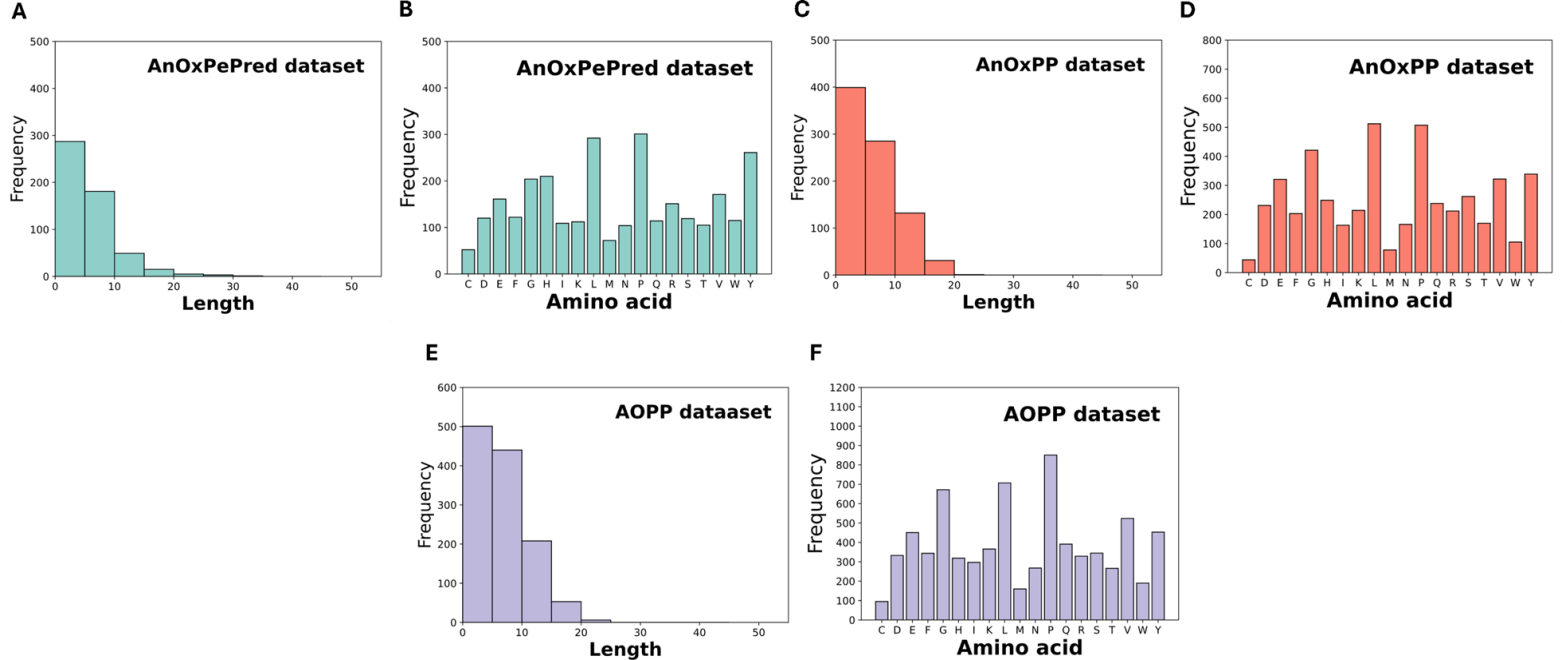
Distribution of AOPs as a function of sequence length and amino acid composition. **A**–**B** for AnOxPePred dataset, **C**–**D** for AnOxPP dataset, **E**–**F** for AOPP dataset

Our analysis of the experimentally validated AOPs in these datasets revealed subtle differences in their distributional patterns. As shown in Fig. 1, the amino acid composition of AOPs in the AnOxPePred dataset exhibited a higher proportion of histidine and a lower proportion of glutamine compared to the other two datasets. In contrast, AOPs the AnOxPP and AOPP datasets demonstrated more similar compositional profiles. These variations underscore the importance of using multiple benchmark datasets to assess the generalizability of models across diverse peptide populations. While the randomly generated negative samples were directly obtained from the three benchmark datasets. This approach is common in bioactive peptide prediction due to the scarcity of experimentally validated non-antioxidant peptides. However, random sequences may be easier to distinguish from AOPs than biologically relevant peptides, potentially leading to optimistic performance estimates.

**Table 1:** Summary of AOP data sets

Dataset	Year	Sources	AOPs	Non-AOPs	Total
AnOxPePred dataset	2020	BIOPEP-UWM	676	728	1404
AnOxPP dataset	2023	DFBP BIOPEP-UWM	1060	1060	2120
AOPP dataset	2025	DFBP BIOPEP-UWM Antimicrobial Peptide database PlantPepDB FermFoodBD	1511	1511	3022

### Proposed Multi-AOP

#### Sequence feature extraction: xLSTM

Peptides are biomolecules composed of amino acids linked by peptide bonds. The sequential nature of these structures has prompted extensive research into sequence models for extracting latent features from peptide primary structures. Long Short-Term Memory (LSTM) networks have proven particularly advantageous in this domain due to their capacity to handle sequential inputs through a memory state mechanism that correlates distinct segments of the sequence (Graves and Graves 2012). Here, we employed xLSTM (Beck et al. 2024), a state-of-the-art special extended LSTM model, to generate robust amino acid sequence features.

The classical LSTM incorporates a memory cell regulated by three gates: the input gate, forget gate, and output gate. At each time step *t*, the forget gate $f_t$ determines the retention rate of previous information stored in memory cell $c_{t-1}$, while the input gate $i_t$ controls the integration of new information $\widetilde{c}_t$ into memory. These operations are defined by the following equations:

1 $$\begin{aligned} f_t= & \sigma (W_f [h_{t-1}, x_t + b_f]), \end{aligned}$$

2 $$\begin{aligned} i_t= & \sigma (W_i [h_{t-1}, x_t + b_i]), \end{aligned}$$

3 $$\begin{aligned} \widetilde{c}_t= & tanh(W_c \cdot [h_{t-1}, x_t] + b_c), \end{aligned}$$

where $h_{t-1}$ represents the output of the previous cell, $x_t$ denotes the current input, and *W* and *b* represent weight matrices and bias vectors, respectively. The sigmoid function $\sigma$ and hyperbolic tangent function $\tanh$ are defined as:

4 $$\begin{aligned} \sigma (x)= & \frac{1}{1 + e^{-x}}, \end{aligned}$$

5 $$\begin{aligned} tanh(x)= & \frac{1 - e^{-2x}}{1 + e^{-2x}}. \end{aligned}$$

The current memory cell state $c_t$ is subsequently updated by integrating retained information from $c_{t-1}$ with new information $\widetilde{c}_t$, enabling selective memory retention or elimination according to:

6 $$\begin{aligned} c_t= f_t \odot c_{t-1} + i_t \odot \widetilde{c}_t. \end{aligned}$$

Finally, the output gate $o_t$ determines which components of the memory state contribute to the current hidden state $h_t$:

7 $$\begin{aligned} o_t= & \sigma (W_o [h_{t-1}, x_t + b_o]) , \end{aligned}$$

8 $$\begin{aligned} h_t= & o_t \odot tanh(c_t), \end{aligned}$$

where $W_o$ and $b_o$ denote the corresponding weight matrix and bias vector.

The xLSTM architecture introduces several significant innovations that address the limitations of classical LSTM. First, it implements an improved gating mechanism for enhanced memory management through scalar LSTM (sLSTM). This mechanism replaces the standard sigmoid function in the input and forget gates with an exponential activation function. To mitigate potential numerical instability from large exponential values, the model stabilizes gates with an additional state $m_t$:

9 $$\begin{aligned} m_t = \max (log(f_t) + m_{t-1}, log(i_t)) . \end{aligned}$$

This exponential gating mechanism enables more dynamic information filtering, improving both information retention and forgetting processes across temporal dependencies. Second, xLSTM introduces matrix LSTM (mLSTM), which replaces traditional scalar memory cells $c \in \mathbb {R}$ with matrix-based memory cells $C \in \mathbb {R}^{d \times d}$. This matrix-based representation supports parallel computation and substantially enhances scalability. The incorporation of a covariance update rule in mLSTM further augments the model’s capacity to efficiently process larger datasets and longer sequences by capturing richer correlation patterns between sequence elements.

Furthermore, xLSTM stacks sLSTM and mLSTM blocks in a residual framework, enhancing model capacity while maintaining computational efficiency. While Transformers have become prevalent in many sequence modeling tasks, xLSTM demonstrates better parameter efficiency, requiring fewer trainable parameters than comparable Transformer models.

#### Graph feature extraction: MPNN

While sequence models like xLSTM excel at modeling the primary structure, graph models can encode information about secondary structural elements and interactions that influence antioxidant properties. MPNNs are a powerful class of graph neural networks (GNNs) designed to process graph-structured data, making them highly suitable for molecular modeling (Gilmer et al. 2020). In the context of our AOP task, AOPs are typically quite short, making it feasible to employ SMILES to represent the atomic and bonding structure of peptides. Then we applied RDKit, a widely used cheminformatics toolkit, to transform the SMILES representations of peptides into molecular graphs (Landrum 2013). In these graphs, atoms are represented as nodes, and the chemical bonds between them are modeled as edges. RDKit further facilitated the extraction of relevant node features and edge features. These features are subsequently fed into the MPNN.

The MPNN operates on graph-structured data by updating node representations through two main stages: the message passing phase and the readout phase. For an input graph data $G=(V, E)$, where each vertex $v \in V$ has an initial feature vector $h^0_v$, and each edge $(v, u) \in E$ may carry an edge feature $e_{vu}$. At each time step *t*, the network uses a message function $M_t$ and an update function $U_t$ to propagate and transform messages across the graph. The message function $M_t$ and update function $U_t$ are defined as:

10 $$\begin{aligned} M(h_v, h_w, e_{vw})= & (h_w, e_{ew}), \end{aligned}$$

11 $$\begin{aligned} U_t(h^t_v, m^{t+1}_v)= & \sigma (H^{deg(v)}_t m^{t+1}_v), \end{aligned}$$

where $(\cdot , \cdot )$ denotes concatenation, $\sigma$ is the sigmoid function, *deg*(*v*) is the degree of vertex *v*, and $H^N_t$ is a learned matrix for each time step *t* and the vertex degree *N*.

The message representation $m^t_v$ is given by

12 $$\begin{aligned} m^{t+1}_v = \sum _{u \in N(v)} M_t(h^t_v, h^t_u, e_{vu}), \end{aligned}$$

the message incorporates the node’s own state $h^t_v$, the neighbor’s state $h^t_u$, and the edge feature $e_{vu}$. Once the message $m^{t+1}_v$ is computed, the node updates its state via the update function $U_t$ resulting in

13 $$\begin{aligned} h^{t+1}_v = U_t(h^t_v, m^{t+1}_v), \end{aligned}$$

The update function modifies the feature representation of each node based on the incoming messages. Through multiple iterations of this process, information can propagate across the molecular graph. After *T* iterations of message passing, a readout function *R* aggregates all final node embeddings into a comprehensive graph representation:

14 $$\begin{aligned} \hat{y}_G = R(\{h^T_v \mid v \in V\}) \end{aligned}$$

The readout function typically employs permutation-invariant operations such as summation or maximization to ensure that the final representation is invariant to node ordering. The final representation encodes the global molecular properties that contribute to antioxidant activity. By leveraging MPNN for graph feature extraction, we enabled our model to capture the complex structural and electronic factors that determine the peptide antioxidant capacity.

#### Model design

**Fig. 2:**
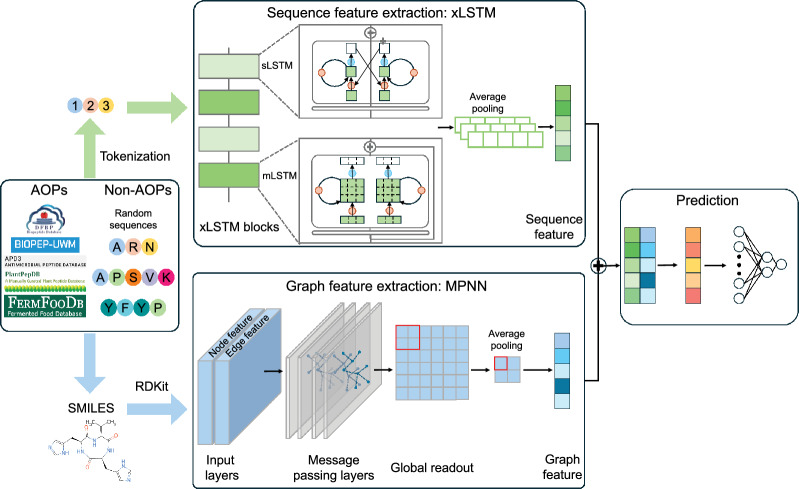
The architecture of Multi-AOP. Multi-AOP comprises two distinct modules. The sequence module employs xLSTM to aggregate the sequence feature from its sequence composition. The graph module employs MPNN to extract the sequence feature from its graph representation

A schematic representation of our proposed Multi-AOP framework is illustrated in Fig. 2. The architecture implements a dual-feature extraction approach, integrating both sequential and structural information to enhance AOP classification performance. For the sequence modeling component, we employed a char-int dictionary to tokenize and pad the sequence to ensure a uniform sequence length. Each input peptide sequence was encoded as a fixed-length integer vector $X = [x_1, x_2,..., x_n]$, where each element $x \in {0, 1,..., 19}$ corresponds to a specific amino acid, and $n = 50$ represents the maximum sequence length. The sequence feature extraction network comprised three stacked xLSTM blocks followed by an average pooling mechanism, yielding a dense embedding vector of dimension of $1 \times 128$.

For structural feature extraction, we implemented a GNN approach to model the two-dimensional molecular topology. The peptide sequences were first converted to SMILES, which were subsequently transformed into molecular graph representations using RDKit (version 2025.09.01). The node and edge features provide a rich representation of the peptide chemical structure. The graph feature extraction network was composed of three message passing layers, a global readout layer, and an average pooling layer. The output extracted graph feature has a dimension of $1 \times 128$.

The integration of sequence and structural information was achieved through a hierarchical fusion of the respective feature vectors. The concatenated feature vector was then processed through two multilayer perceptrons (MLPs), each followed by a dropout regularization layer, facilitating information exchange between the complementary feature spaces. This multimodal integration enabled the model to leverage both the sequential patterns and structural motifs characteristic of antioxidant peptides.

To determine the optimal model configuration, we conducted comprehensive hyperparameter optimization through grid search. Our optimization strategy proceeded in two phases: first, we independently evaluated the performance of the xLSTM and graph model components, empirically establishing that three xLSTM blocks and three MPNNConv layers provided optimal feature extraction capabilities. Subsequently, we fine-tuned the critical hyperparameters of the fusion module, exploring various integration strategies and regularization parameters. The hyperparameter search space encompassed fusion methodologies, learning rate, weight decay rate, and dropout rate, with performance metrics summarized in Table 2. Model training was performed using the Adam optimizer with binary cross-entropy as the objective function. The final optimized architecture incorporated three xLSTM blocks and three MPNNConv layers, with a learning rate of 1e-5, a dropout rate of 2e-1, weight decay of 3e-3, and a batch size of 64.

**Table 2:** Hyperparameters and candidate value ranges considered during model optimization

Hyperparameter	Candidate value	Optimal value
Fusion strategies	concatenation, cross attention, hierarchical	hierarchical
Number of FC layers	1, 2, 3, 4	3
Weight decay rate	1e-2, 3e-2,... 1e-3, 3e-3,...,7e-3	3e-3
Dropout rate	1e-1, 2e-1, 3e-1, ..., 9e-1	2e-1
Learning rate	1e-4, 1e-5, 1e-6	1e-5

### Model performance metrics

We used four general performance metrics: Mathews correlation coefficient (MCC), accuracy, precision, and sensitivity. These metrics are calculated through four metrics: true positive (TP), true negative (TN), false positive (FP), and false negative (FN).

15 $$\begin{aligned} MCC= & \frac{TP \times TN - FP \times FN}{\sqrt{(TP + FP) (TP +F N)(TN + FP)(TN + FN)}} \end{aligned}$$

16 $$\begin{aligned} Accuracy= & \frac{TP + TN}{TP + TN + FP + FN} \end{aligned}$$

17 $$\begin{aligned} Precision= & \frac{TP}{TP + FP} \end{aligned}$$

18 $$\begin{aligned} Sensitivity= & \frac{TP}{TP + FN} \end{aligned}$$

19 $$\begin{aligned} Specificity= & \frac{TN}{TN + FP} \end{aligned}$$

## Results

### Sequence model selection

To evaluate the efficacy of xLSTM for AOP prediction, we first compared its performance with Transformer-based protein language models (PLMs) such as ProtBERT (Elnaggar et al. 2021) and ESM2 (Lin et al. 2022). PLMs are known for their ability to capture long-range dependencies through self-attention mechanisms. PLMS have shown to be highly effective for tasks that require modeling complex structural and functional relationships in protein sequences. However, these models come with a significant drawback: their massive parameter sizes, often in the hundreds of millions. This large number of parameters makes PLMs prone to overfitting when applied to specialized tasks with limited data, such as AOP prediction.

In contrast, the xLSTM model offers a more parameter-efficient solution, containing only 0.70 million parameters–just 0.17% and 0.47% of the parameter sizes of ProtBERT and ESM2, respectively. With its substantially reduced parameters, the proposed xLSTM model achieved consistently competitive performance across all three benchmark datasets. Specifically, xLSTM attained classification accuracies of 0.7879, 0.9375, and 0.8804 on the AnOxPePred, AnOxPP, and AOPP datasets, respectively. This result demonstrates that xLSTM can provide an effective framework for modeling antioxidant peptide sequences without the risk of overfitting inherent in large-scale PLMs.

To ensure an accurate estimate of the performance of various models, we split each benchmark dataset into five partitions for cross-validation. The test was performed independently for each fold, using the remaining folds for training. The average performance of the test folds was reported. These comparisons are summarized in Table 3. Experimental results show that the xLSTM’s parameter-efficient design, advanced gating mechanisms, and specialized architecture make it a more effective and scalable solution for AOP prediction.

Moreover, we compared the xLSTM model to a vanilla multi-layer LSTM. The exponential gating mechanism and matrix-based memory cells in xLSTM offer significant improvements in feature extraction, even for short peptide sequences, where standard LSTMs are typically considered sufficient. As a result, xLSTM outperformed the vanilla LSTM across all three datasets, further underscoring its superior ability to predict AOPs.

**Table 3:** Comparative results of the five-fold CV of ProtBERT, ESM2, and xLSTM sequence models across the three benchmark datasets

Dataset	Model	Number of parameters	Accuracy	Precision	Sensitivity	Specificity	MCC
AnOxPePred dataset	ProtBERT	420 M	0.7198	0.7683	0.6000	0.8699	0.4493
	ESM2	150 M	0.7858	0.7855	0.7630	0.8151	0.5710
	LSTM	0.63 M	0.7621	0.7729	0.7120	0.8048	0.5205
	xLSTM	0.70 M	0.7879	0.7957	0.7526	0.8093	0.5755
AnOxPP dataset	ProtBERT	420 M	0.9292	0.9441	0.9132	0.9481	0.8592
	ESM2	150 M	0.9340	0.9646	0.9009	0.9128	0.8698
	LSTM	0.63 M	0.9187	0.9279	0.9297	0.9037	0.8238
	xLSTM	0.70 M	0.9375	0.9483	0.9257	0.9537	0.8754
AOPP dataset	ProtBERT	420 M	0.8769	0.9232	0.8224	0.9340	0.7585
	ESM2	150 M	0.8746	0.9188	0.8218	0.9140	0.7534
	LSTM	0.63 M	0.8710	0.9095	0.8289	0.9027	0.7336
	xLSTM	0.70 M	0.8804	0.9168	0.8366	0.9359	0.7637

### Graph model selection

Graph Neural Networks (GNNs) have emerged as sophisticated tools for feature extraction from graph-structured data, particularly for molecular representations where nodes correspond to atoms and edges represent chemical bond interactions (Dauparas et al. 2022; Sumida et al. 2024). The relatively constrained length of AOPs enables effective SMILES transformation into molecular graphs without computational overhead, making graph-based approaches particularly suitable for this domain. Here, we examined five prominent GNN architectures: GraphSAGE (Hamilton et al. 2017), Graph Attention Networks (GATs) (Velickovic et al. 2017), Graph Isomorphism Networks (GINs) (Xu et al. 2018), MPNNs, and ChebNet (Tang et al. 2024). Each of these frameworks has previously demonstrated efficacy in modeling complex relationships between molecular structures and their biological activities through distinct computational mechanisms.

As mentioned, MPNNs operate through an iterative information exchange protocol wherein nodes update their representations by aggregating messages from neighboring nodes. This process enables the incorporation of both local chemical environment information and, through multiple iterations, global structural patterns. In contrast, GraphSAGE generates node embeddings by sampling and aggregating features from local neighborhoods, offering scalability advantages for large molecular graphs. GATs implement an attention-based mechanism that differentially weights neighboring nodes according to their contextual relevance. This adaptive focus allows GATs to prioritize particularly informative atomic interactions within the peptide structure, potentially highlighting functional groups critical for antioxidant activity. GINs, meanwhile, employ more mathematically expressive aggregation functions designed to enhance discriminative capabilities between isomorphically distinct graph structures–a property particularly valuable for precise molecular fingerprinting of peptides with similar compositions but different arrangements. ChebNet, proposed in 2024, utilizes spectral graph convolutions based on Chebyshev polynomials to capture multi-scale structural information across molecular topologies.

Table 4 presents a comprehensive performance analysis of these GNN architectures across our three benchmark datasets. Consistent with the experimental protocol described above, all models were evaluated using five-fold cross-validation on the three benchmark datasets. The MPNN architecture exhibited the highest overall performance among all evaluated graph models. The model achieved accuracies of 0.7473, 0.8703, and 0.8201 on the AnOxPePred, AnOxPP, and AOPP datasets, respectively. This performance can be attributed to MPNN’s effective balance between local chemical environment modeling and global structural pattern recognition capabilities in identifying the molecular motifs associated with antioxidant capacity in peptides.

**Table 4:** Comparative results of the five-fold CV of MPNN, GAT, and GIN graph neural models across the three benchmark datasets.

Dataset	Model	Number of parameters	Accuracy	Precision	Sensitivity	Specificity	MCC
AnOxPePred dataset	GraphSAGE	23 k	0.7395	0.7372	0.7037	0.7739	0.4440
	GAT	54 k	0.7331	0.7055	0.7630	0.6300	0.4684
	GIN	19 k	0.7082	0.7387	0.6074	0.7242	0.4178
	ChebNet	32 k	0.7357	0.7344	0.7274	0.7764	0.4674
	MPNN	75 k	0.7473	0.7462	0.7185	0.7871	0.4935
AnOxPP dataset	GraphSAGE	23 k	0.8392	0.8634	0.8718	0.8347	0.7388
	GAT	54 k	0.8160	0.7557	0.9340	0.8092	0.6504
	GIN	19 k	0.6651	0.6232	0.8349	0.8007	0.3511
	ChebNet	32 k	0.8199	0.8310	0.8500	0.8299	0.7355
	MPNN	75 k	0.8703	0.8489	0.9009	0.8396	0.7420
AOPP dataset	GraphSAGE	23 k	0.8178	0.8286	0.8033	0.8323	0.6368
	GAT	54 k	0.7525	0.6997	0.8845	0.8408	0.5235
	GIN	19 k	0.6452	0.6215	0.7426	0.7267	0.2961
	ChebNet	32 k	0.8131	0.8334	0.7676	0.8407	0.6330
	MPNN	75 k	0.8201	0.8440	0.7855	0.8548	0.6418

### Comparative analysis of baseline prediction models

To establish benchmark performance, we conducted experiments using different machine learning (ML) and deep learning (DL) algorithms, in combination with various peptide feature encoding methods. For conventional ML algorithms, we selected five widely-used classifiers that represent diverse learning paradigms: Decision Tree (DT), K-Nearest Neighbors (KNN), Support Vector Machine (SVM), Random Forest (RF), and eXtreme Gradient Boosting (XGBoost). DT was included as a simple, interpretable baseline. KNN represents instance-based learning that classifies samples based on similarity in feature space. SVM was selected for its effectiveness in high-dimensional spaces and its proven performance in peptide classification tasks. RF and XGBoost represent ensemble methods that combine multiple weak learners, with XGBoost being particularly effective for structured data. These algorithms were evaluated with twelve peptide descriptors commonly used in peptide property prediction, including Amino Acid Composition (AAC), Dipeptide Composition (DPC), Composition-Transition-Distribution (CTD), and Dipeptide Deviation from Expected Mean (DDE). A complete list of descriptors is provided in Supplementary Table S1.

In addition to conventional ML approaches, we included AnOxPePred, AOPP, and PepBERT, which are publicly available methods specifically developed for peptide property prediction. AnOxPePred employs one-hot encoding with convolutional neural networks (CNNs), representing a standard sequence-based approach. AOPP utilizes bidirectional LSTM (Bi-LSTM) with fused amino acid descriptors, representing a more advanced architecture that captures bidirectional sequential dependencies. PepBERT (Zhenjiao et al. 2025) is a BERT-based model pre-trained on peptide sequences from UniRef. Following its original implementation, we utilized PepBERT as a feature extractor to generate sequence embeddings and trained a logistic regression classifier for AOP prediction. It should be noted that the AnOxPP model was excluded from our comparative analysis due to the unavailability of its source code for replication.

Table 5 presents the comprehensive performance metrics of various ML and DL model-descriptor combinations across the three benchmark datasets. Interestingly, the combination of the SVM classifier with DDE descriptors demonstrated efficacy, surpassing the specialized DL models on the AnOxPePred and AnOxPP dataset. The SVM model achieved an accuracy of 0.7379 on the AnOxPePred dataset and an accuracy of 0.9528 on the AnOxPP dataset. The p-values from paired t-tests comparing the accuracy of the SVM models and our proposed Multi-AOP model are both less than 0.001 on the AnOxPePred, AnOxPP datasets, indicating that the improvement of Multi-AOP is statistically significant.

Among the evaluated specialized DL frameworks, AOPP consistently outperformed AnOxPePred across all three benchmark datasets. This performance gain can be attributed to its capability of Bi-LSTM architectures to capture bidirectional sequential dependencies in peptide sequences compared to the conventional CNNs. Compared to the next best DL method AOPP, our proposed Multi-AOP framework achieved further improvements in classification accuracies, reaching 0.8043 ($\uparrow$9.00%), 0.9684 ($\uparrow$1.64%), and 0.9043 ($\uparrow$ 0.14%) on the AnOxPePred, AnOxPP, and AOPP datasets, respectively. The improvements in accuracy are statistically significant, as indicated by p-values below the 0.05 significance level (6.453e-5, 2.442e-03, and 0.0486, respectively). This further performance gain stemmed from the multi-view learning strategy, which enables the model to capture complementary features from both primary sequence and molecular graph representations, resulting in more robust and generalizable predictions of peptide antioxidant activity.

**Table 5:** Comparative results of the five-fold CV of Multi-AOP with various machine learning algorithms and existing deep learning prediction models across the three benchmark datasets

Dataset	Model	Feature	Number of parameters	Accuracy	Precision	Sensitivity	Specificity	MCC
AnOxPePred dataset	DT	AAC	-	0.6726	0.6503	0.6889	0.6575	0.3462
	KNN	DDE	-	0.7260	0.7101	0.6909	0.7260	0.4517
	RF	AAC	-	0.6940	0.6606	0.5950	0.7688	0.3697
	SVM	DDE	-	0.7379	0.7448	0.6917	0.7814	0.4745
	XGBoost	AAC	-	0.7331	0.7143	0.6741	0.7534	0.4654
	AnOxPePred	one-hot encoding	38 k	0.6441	0.6788	0.6908	0.6703	0.3506
	AOPP	ADCA^a^	0.18 M	0.7315	0.7160	0.7361	0.7269	0.4652
	PepBERT	embedding layer	4.94 M	0.7580	0.7680	0.7111	0.8014	0.5152
	Multi-AOP	embedding layer	0.75 M	0.8043	0.8226	0.7556	0.8493	0.6086
AnOxPP dataset	DT	AAC	-	0.9033	0.9091	0.8962	0.9040	0.8067
	KNN	AAC	-	0.9033	0.9296	0.8726	0.9340	0.8081
	RF	AAC	-	0.9316	0.9552	0.9057	0.9717	0.8644
	SVM	DDE	-	0.9528	0.9798	0.9151	0.9806	0.9083
	XGBoost	AAC	-	0.8679	0.8611	0.8774	0.8585	0.7360
	AnOxPePred	one-hot encoding	38 k	0.9316	0.9420	0.9198	0.9434	0.8634
	AOPP	ADCA	0.18 M	0.9410	0.9579	0.9237	0.9592	0.8837
	PepBERT	embedding layer	4.94 M	0.9245	0.9369	0.9104	0.9387	0.8494
	Multi-AOP	embedding layer	0.75 M	0.9684	0.9835	0.9528	0.9839	0.9373
AOPP dataset	DT	AAC	-	0.8020	0.7020	8020	0.8185	0.6040
	KNN	AAC	-	0.8465	0.8860	0.7954	0.8977	0.6967
	RF	AAC	-	0.8629	0.9065	0.8317	0.9076	0.7484
	SVM	CTDC	-	0.8878	0.9608	0.8086	0.9670	0.7855
	XGBoost	AAC	-	0.8383	0.8571	0.8119	0.8713	0.6775
	AnOxPePred	one-hot encoding	38 k	0.8762	0.9403	0.7987	0.9538	0.7617
	AOPP	ADCA	0.18 M	0.9030	0.9422	0.8607	0.9456	0.8103
	PepBERT	embedding layer	4.94 M	0.8729	0.9124	0.8251	0.9208	0.7493
	Multi-AOP	embedding layer	0.75 M	0.9043	0.9646	0.8396	0.9690	0.8156

^a^ADCA: feature concatenation of Amino Acid Composition (AAC), Dipeptide Composition (DPC), Compositionof K-Spaced Amino Acid Group Pairs (CKS), and Amino Acid Index (AAI).

### Ablation study

**Fig. 3:**
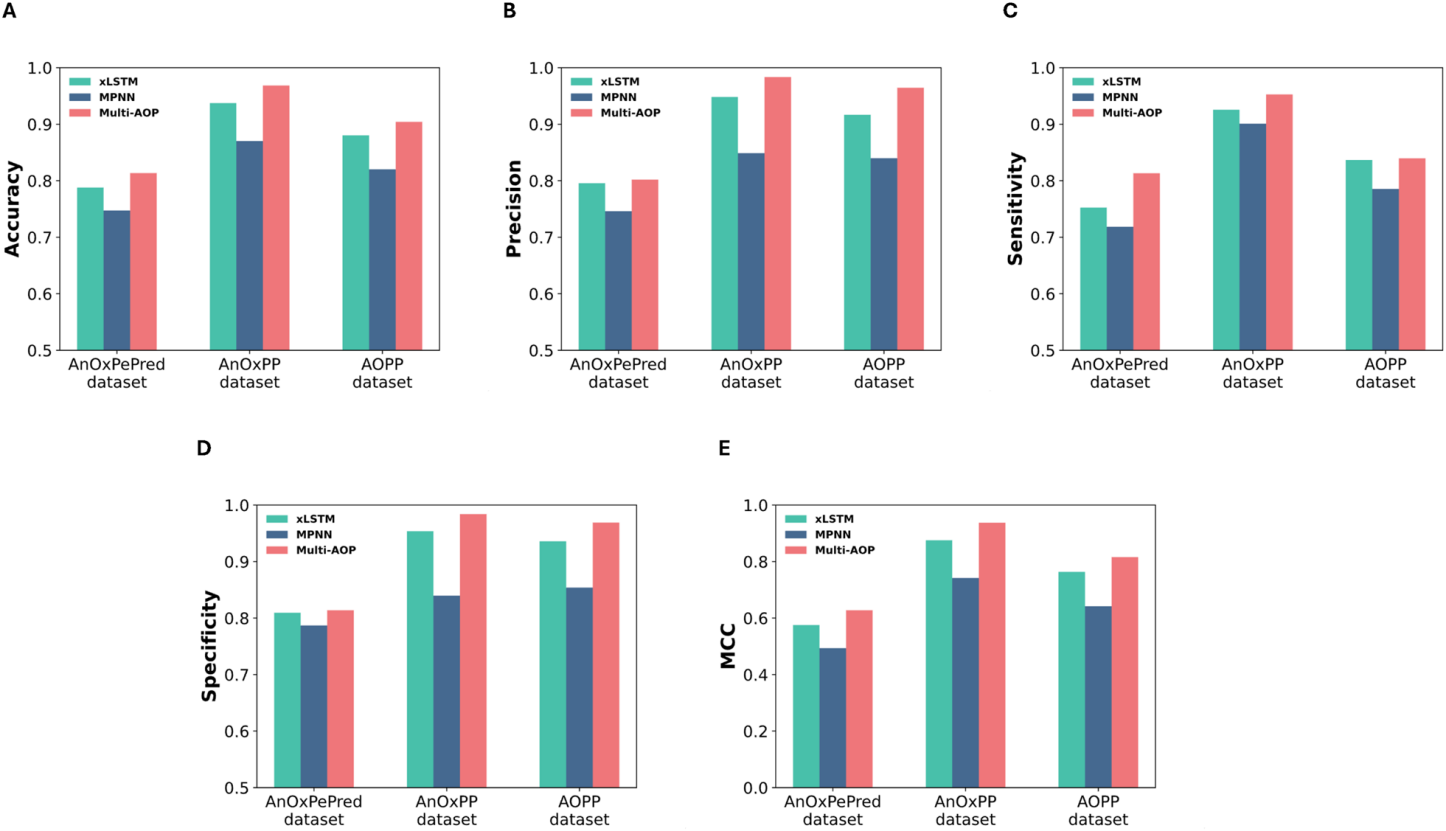
Ablation study. Performance comparison of Multi-AOP and its sub-models in predicting peptide antioxidant activity

Our Multi-AOP framework complemented primary sequence features extracted from the xLSTM sequence model with two-dimensional structural features from the MPNN graph model. To evaluate the contribution of each component, we conducted an ablation study. As shown in Fig. 3, we compared the performance of individual sub-models against our integrated approach across multiple metrics on all benchmark datasets. The sequence model consistently outperformed the graph model. However, our hybrid model achieved substantial performance improvements over both individual sub-models, demonstrating that the structural information encoded in two-dimensional molecular graphs provides complementary insights that enhance the predictive capability of sequence-based embeddings alone.

To further investigate the relative contribution of sequence features and structural features across different types of peptides, we categorized peptide sequences into short peptides ($<15$ amino acids) and long peptides ($\ge 15$ amino acids). This threshold represents the minimum length needed for stable secondary structure formation, as $\alpha$-helices typically require 10–15 residues to stabilize. We hypothesize that because short peptides remain structurally flexible, their antioxidant activity is primarily determined by their amino acid composition. In contrast, longer peptides can form folded structures in which the spatial positioning of reactive groups becomes critical for their radical scavenging function. The test was performed on the merged dataset of the three benchmark data, yielding a dataset of 5,235 peptides, comprising 2,597 AOPs and 2,638 random sequences.

As shown in Table 6, the xLSTM-based sequence model achieved a higher accuracy of 0.8607 on short peptides, while the MPNN-based graph model performed better on long peptides with an accuracy of 0.8571. This indicates that sequence features dominate in shorter peptides, whereas structural features become more important as peptide length increases. Of note, our Multi-AOP framework showed reduced performance on long peptides. This performance gap may be attributed to the limitations of SMILES-derived molecular graphs for longer peptides, where the increased graph complexity introduces noise that can obscure relevant structural patterns.

**Table 6:** Ablation study of Multi-AOP by peptide length on the merged dataset.

Sequence length	Model	Accuracy	Precision	Sensitivity	Specificity	MCC
Short peptides ($< 15$ aa)	xLSTM	0.8607	0.8535	0.8566	0.8449	0.7215
	MPNN	0.7901	0.7840	0.7967	0.7836	0.5803
	Multi-AOP	0.8778	0.8569	0.8828	0.8528	0.7360
Long peptides ( $>15$ aa)	xLSTM	0.7321	0.7333	0.7586	0.7037	0.4632
	MPNN	0.8571	0.8529	0.9063	0.7917	0.7072
	Multi-AOP	0.8214	0.8065	0.8621	0.7778	0.6431

### External validation on newly curated data

**Fig. 4:**
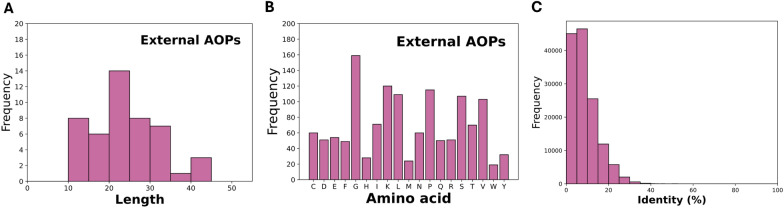
Performance evaluation of Multi-AOP on the newly discovered AOPs: **A** Distribution of sequence length, **B** Distribution of sequence amino acid composition, **C** Distribution of sequence identity between the combined dataset and the external dataset

To enhance the practical utility of our proposed model, we developed the final prediction model trained on the whole merged dataset containing 5,235 sequences. Furthermore, we evaluated the performance of models trained on individual datasets versus the merged one, while the 54 newly published AOPs were used as the external validation set. These 54 newly published AOPs collected from diverse sources including DEBP, BIOPEP-UWM database, Antimicrobial Peptide database, Plant-PepDB, and FermFooDB. The distributions of the length and amino acid composition are shown in Fig. 4 A and B. Note that these external sequences demonstrated minimal sequence homology with our training data, exhibiting an average sequence identity below 9%, as illustrated in Fig. 4 C. As presented in Table 7, our final model trained on the merged dataset achieved an impressive sensitivity of 0.9057 on this challenging external dataset, highlighting the benefits of data integration. While AnOxPePred and AOPP obtained sensitivities of 0.5849 and 0.7380, these performance comparison closely aligned with the results obtained on the test sets of the three benchmark datasets, providing evidence of the generalizability of our final AOP prediction model.

**Table 7:** External validation performance of Multi-AOP trained on individual and merged datasets

Validation set	Training data	Number of sequences	Sensitivity
54 newly published AOPs	AnOxPePred dataset	1404	0.5340
	AnOxPP dataset	2120	0.8802
	AOPP dataset	3022	0.8697
	Merged dataset	5235	0.9057

## Discussion and conclusion

In this work, we proposed Multi-AOP, a novel framework that integrates multi-view information to predict peptide antioxidant activity, achieving state-of-the-art accuracy on three benchmark datasets. Our approach leverages the strengths of both sequence-based and structure-based representations, demonstrating that hierarchical fusion of diverse molecular features can enhance predictive performance. Compared with pre-trained PLMs containing hundreds of millions of parameters, we designed a parameter-efficient xLSTM architecture to extract sequence features from amino acid sequences. Additionally, the intrinsically short length of AOPs makes it feasible to convert peptide sequences into SMILES representations, which enable the construction of molecular graphs for training GNNs to learn two-dimensional structural features. The sequence and graph features were then hierarchically fused for AOP prediction.

Our Multi-AOP framework shares conceptual similarities with several recently developed hybrid peptide prediction methods. Multi-Peptide combines PeptideBERT with Graph Neural Networks using a CLIP-inspired contrastive loss framework, achieving an accuracy of 88.057% (Badrinarayanan et al. 2024). However, this approach relies on the computationally expensive PeptideBERT backbone (420 million parameters). In contrast, Multi-AOP (0.75 million parameters) employs a lightweight xLSTM-based sequence encoder combined with SMILES-derived 2D molecular graphs.

Comparative evaluation with ML and DL prediction models demonstrated that our proposed model outperformed both traditional ML models and existing DL architectures. Specifically, Multi-AOP achieved accuracies of 0.8043, 0.9684, and 0.9043 on the AnOxPePred, AnOxPP, and AOPP datasets, respectively. To facilitate broader adoption, we trained a final prediction model on the merged dataset combining all three benchmarks. External validation confirmed the generalizability of this model on newly published AOP sequences. Beyond its predictive performance, Multi-AOP offers considerable practical value for in silico screening of large-scale peptide libraries, accelerating the identification of antioxidant peptides for drug discovery and nutraceutical development.

While our current framework successfully integrates sequence and two-dimensional structural information, several limitations warrant consideration. First, the model does not explicitly handle post-translational modifications (PTMs), as the training data primarily consist of unmodified peptide sequences. Addressing this limitation would require datasets containing modified residues and suitable molecular representations. Second, our SMILES-to-graph conversion via RDKit may fail for non-standard amino acids or unusual peptide structures, potentially limiting its applicability to unconventional sequences.

Furthermore, our ablation study revealed that Multi-AOP performs better on short peptides ($<15$ amino acids) than on longer peptides ($\ge 15$ amino acids). This performance gap suggests that two-dimensional graph representations are insufficient to capture the conformational complexity of longer peptides, where antioxidant activity may depend on three-dimensional spatial arrangements of functional groups. Recent advances in AI-based protein structure prediction, particularly AlphaFold (Bryant et al. 2022) and ESMFold (Lin et al. 2022), have made high-quality three-dimensional structural information readily accessible. In future work, we plan to integrate predicted 3D coordinates as additional node features in the graph representation to capture spatial relationships between atoms.

Finally, we aim to incorporate explainability techniques such as SHapley Additive exPlanations (SHAP) (Lundberg and Lee 2017) or attention visualization to identify key amino acids and structural motifs contributing to antioxidant activity. Such interpretable insights could guide rational peptide design by revealing sequence-activity relationships and highlighting functional residues critical for radical scavenging. Looking forward, Multi-AOP provides a foundation for sustainable peptide discovery by enabling rapid, resource-efficient screening of candidate AOPs. As experimental AOP databases continue to expand, retraining Multi-AOP on larger and more diverse datasets will further improve its predictive accuracy and generalizability. We envision Multi-AOP contributing to the development of next-generation natural antioxidants for food preservation, pharmaceutical formulation, and therapeutic applications.

## Supplementary Information

Supplementary material 1.

## Data Availability

All peptide data used in this study was obtained from the DEBP (http://www.cqudfbp.net/index.jsp), BIOPEP-UWM database (https://biochemia.uwm.edu.pl/biopep/start_biopep.php), Antimicrobial Peptide database (https://aps.unmc.edu/), PlantPepDB (http://14.139.61.8/PlantPepDB/), and FermFooDb (https://webs.iiitd.edu.in/raghava/fermfoodb/). The data and the optimized model can be downloaded from https://github.com/CaiJianxiu/Multi-AOP.
