# Supplementary material for: Multi-AOP: a lightweight multi-view deep learning framework for antioxidant peptide discovery
Source: Bioresour Bioprocess. 2026 Feb 2;13(1):21. doi: 10.1186/s40643-025-01004-1 (PMC12864603; doi:10.1186/s40643-025-01004-1)
Supplement: Supplementary file 1 — Supplementary material 1. [file 40643_2025_1004_MOESM1_ESM.pdf]

Multi-AOP: A Lightweight Multi-View Deep Learning Framework for Antioxidant Peptide Discovery

Jianxiu Cai,[1,2,†] Xinpo Lou, [1,3,†] Chak Fong Chong, [1,2] Deepa Alex, [4]
Joel P. Arrais, [2,*] Yapeng Wang,[1,*] Shirley W. I. Siu[1,3,*]

[1]Faculty of Applied Sciences,
Macao Polytechnic University, Rua de Luís Gonzaga Gomes, Macau SAR, China
[2]Department of Informatics Engineering,
University of Coimbra, Paço das Escolas, Coimbra, Portugal
[3]Centre for Artificial Intelligence Driven Drug Discovery,
Macao Polytechnic University, Rua de Luís Gonzaga Gomes, Macau SAR, China
[4]BioMyne-Biotech Innovation and Engineering Ltd
[†]These authors contributed equally to this work.
[*]To whom correspondence should be addressed;
E-mail: jpa@dei.uc.pt; yapengwang@mpu.edu.mo; shirleysiu@mpu.edu.mo.

Table S1: Abbreviation of peptide feature descriptors

Abbreviation	Descriptor Full Name
AAC	Amino Acid Composition
CTD-C	Composition
CTD-D	Transition
CTD-T	Distribution
CTriad	Conjoint Triad
DDE	Dipeptide Deviation from Expected Mean
GAAC	Grouped Amino Acid Composition
DPC	Di-Peptide Composition
GDPC	Grouped Di-Peptide Composition
GTPC	Grouped Tri-Peptide Composition
KSCTriad	k-Spaced Conjoint Triad
QSOrder	Quasi-sequence-order

Table S2: Ablation study of Multi-AOP across the three benchmark datasets

Dataset	Model	Accuracy	Precision	Sensitivity	Specificity	MCC
AnOxPePred dataset	xLSTM	0.7879	0.7957	0.7526	0.8093	0.5755
	MPNN	0.7473	0.7462	0.7185	0.7871	0.4935
	Multi-AOP	**0.8043**	**0.8226**	**0.7556**	**0.8493**	**0.6086**
AnOxPP dataset	xLSTM	0.9375	0.9483	0.9257	0.9537	0.8754
	MPNN	0.8703	0.8489	0.9009	0.8396	0.7420
	Multi-AOP	**0.9684**	**0.9835**	**0.9528**	**0.9839**	**0.9373**
AOPP dataset	xLSTM	0.8804	0.9168	0.8366	0.9359	0.7637
	MPNN	0.8201	0.8400	0.7855	0.854	0.6418
	Multi-AOP	**0.9043**	**0.9646**	**0.8396**	**0.9690**	**0.8156**
